# Supplementary material for: Factors associated with health-seeking behavior amongst children in the context of free market: Household study in Ouagadougou, Burkina Faso, 2011
Source: PLoS One. 2022 Oct 18;17(10):e0271493. doi: 10.1371/journal.pone.0271493 (PMC9578640; doi:10.1371/journal.pone.0271493)
Supplement: S1 Table — (DOCX) [file pone.0271493.s002.docx]

**S1:** Multilevel analysis using Poisson regression^Ø^ for the choice between the formal providers by urban children by health condition

| Individual characteristics | Emergency | | | | Severe* | | | | Non-severe | | | |
| --- | --- | --- | --- | --- | --- | --- | --- | --- | --- | --- | --- | --- |
|  | FP vs. Public | | NFP vs. Public | | FP vs. Public | | NFP vs. Public | | FP vs. Public | | NFP vs. Public | |
|  | OR | 95%CI | OR | 95%CI | OR | 95%CI | OR | 95%CI | OR | 95%CI | OR | 95%CI |
| Age, year |  |  |  |  |  |  |  |  |  |  |  |  |
| 5-14 | 1 |  | 1 |  | 1 |  | 1 |  | 1 |  | 1 |  |
| <1 | .088 | -.495; .319 | .166 | -.159; .492 | -.014 | -.360; .331 | .087 | -.248; .424 | .333 | -.145; .812 | .346 | -.128; .822 |
| 1-4 | -.088 | -.288; .112 | -.074 | -.274; .126 | -.022 | -.208; .163 | -.023 | -.220; .172 | -.153 | -.452; .144 | -.037 | -.334; .258 |
| Gender |  |  |  |  |  |  |  |  |  |  |  |  |
| Boy | 1 |  | 1 |  | 1 |  | 1 |  | 1 |  | 1 |  |
| Girl | .043 | -.124; .211 | -.046 | -.210; .116 | .048 | -.108; 204 | -.012 | -.175; .149 | .090 | -.336; .154 | -.185 | -.420; .048 |
| Filiation |  |  |  |  |  |  |  |  |  |  |  |  |
| Other children | 1 |  | 1 |  | 1 |  | 1 |  | 1 |  | 1 |  |
| Headship’s Son-D\|grdSon-D | .227 | -.178; .633 | .005 | -.367; .377 | -.274 | -.583; .034 | -.181 | -.541; .178 | .387 | -.245; 1.020 | -.411 | -.906; .084 |
| Insurance |  |  |  |  |  |  |  |  |  |  |  |  |
| Not Insured | 1 |  | 1 |  | 1 |  | 1 |  | 1 |  | 1 |  |
| Insured | -.461 | -.797;-.125 | -.508 | -.964;-.052 | -1.05 | -1.33;-.786 | -.408 | -.849; .026 | -.778 | -1.16; -.388 | -.752 | -1.318;-.186 |
| Household Characteristics |  |  |  |  |  |  |  |  |  |  |  |  |
| Household size |  |  |  |  |  |  |  |  |  |  |  |  |
| >3 children | 1 |  | 1 |  | 1 |  | 1 |  | 1 |  | 1 |  |
| 1 child | .029 | -0.23; .298 | .144 | -.121; .409 | .285 | .0355; .535 | .136 | -.138; .410 | .446 | .032; .859 | .086 | -.298; .472 |
| 2-3 children | -.027 | -.230; .174 | .060 | -.140; .261 | .119 | -.073; .312 | .196 | -.010; .402 | .442 | .118; .765 | -.006 | -.295; .281 |
| Headship’s head age, year | .011 | .0028; .019 | -.001 | -.009; .006 | -.010 | -.017;-.003 | -.009 | -.017;-.001 | .010 | -.002; .023 | .012 | .001; .023 |
| Headship’s gender |  |  |  |  |  |  |  |  |  |  |  |  |
| Men | 1 |  | 1 |  | 1 |  | 1 |  | 1 |  | 1 |  |
| Women | .388 | .016; .760 | -.131 | -.439; .176 | .070 | -.236;.376 | -.080 | -.389; .228 | -.779 | -1.673; .115 | -.085 | -.714; .543 |
| Headship’s marital status |  |  |  |  |  |  |  |  |  |  |  |  |
| In union | 1 |  | 1 |  | 1 |  | 1 |  | 1 |  | 1 |  |
| Otherwise | -.421 | -.883; .411 | .094 | -.274; .464 | -.480 | -.901;-.059 | -.158 | -.541; .225 | .130 | .753; 1.053 | -.187 | -.908; .532 |
| Headship’s education |  |  |  |  |  |  |  |  |  |  |  |  |
| No formal education | 1 |  | 1 |  | 1 |  | 1 |  | 1 |  | 1 |  |
| Primary | -1.10 | -1.40;-.805 | -.121 | -.459; .216 | - | - | -.069 | -.407; .268 | 1.17 | -1.641;-.705 | .597 | .124; 1.071 |
| Secondary | -.520 | -.846;-.194 | -.129 | -.501; .241 | - | - | .052 | -.311; .145 | -.913 | -1.476;-.351 | -.072 | -.670; .526 |
| University | -.542 | -.791;-.293 | -.056 | -.357; .244 | - | - | -.316 | -.611;-.020 | -.289 | -.627; .048 | .474 | .043; .905 |
| Headship’s employment |  |  |  |  |  |  |  |  |  |  |  |  |
| Not in labor | 1 |  | 1 |  | 1 |  | 1 |  | 1 |  | 1 |  |
| Government job | -.343 | -.667;.-020 | .023 | -.308; .355 | - | - | -.122 | -.450; .205 | .251 | -.749; .247 | -.323 | -.858; .211 |
| Parapub & formal private | -.287 | -.564;-.009 | -.125 | -.445; .194 | - | - | -.155 | -.470; .158 | -.449 | -.807; -.091 | -.429 | -.885; .027 |
| Informal private | -.145 | -.412; .120 | .134 | -.142; .413 | - | - | -.146 | -.420; .128 | -.276 | -.670; .118 | -.173 | -.585; .238 |

^Ø^The iteration completes only when implementing Poisson regression

*The iteration completes when the variables ‘Headship’s education’ and ‘Headship’s employment’ were removed.
