# Supplementary material for: Factors associated with health-seeking behavior amongst children in the context of free market: Household study in Ouagadougou, Burkina Faso, 2011
Source: PLoS One. 2022 Oct 18;17(10):e0271493. doi: 10.1371/journal.pone.0271493 (PMC9578640; doi:10.1371/journal.pone.0271493)
Supplement: Attachment — Submitted filename: Reviewers comments.LAST-mars2020.docx [file pone.0271493.s003.docx]

PONE-D-18-15646R1
Urban children health-seeking-behaviors in the context of free market: Household study in Ouagadougou, Burkina Faso
PLOS ONE

**Review Comments to the Author


Reviewer #1:**

**1.1:** My previous comments were not addressed. In the way questions were asked to participants ("where do you seek care in case of ..."), the construct validity is a major issue.

*We thank the reviewer for the comment.*

*A structured questionnaire was developed in line with a set of former questionnaires designed and used in Burkina Faso Demographic and Health Survey (DHS). Prior to this, we conveyed three academics (NH, CC-Y, LC-Y) and one field researcher (MKD) to conduct a content validity check of the candidate questionnaire. Subsequently, forward and backward translations into French were conducted. Finally, the questionnaire was pilot-tested using 32 households from four Administrative Sectors (AS) in the study setting to perform semantic adjustments.*

*The questions are framed such as: Where does [name of the household member] seek care in case of a health emergency? We believe this formulation is not in a hypothetical perspective. Instead, we sought their usual health-care-seeking behavior. Finally, the question “Could you tell me if you have suffered from any disease/injury in the past 30 days?” was posed toward the end in order to validate their early statement.*

**1.2:** Authors rebutted our comment and stated that, after consulting several biostatisticians, "they do not have a reasonable statistical foundation to implement a multilevel analysis" because "people from all characteristics, including income level, ethnicity, profession... are mixed up in all the administrative sectors of the city". We disagree. First, the statistical foundation is that the structure of the data is hierarchical. Second, what test was performed that make the authors say that participants are mixed up in all the administrative sectors of the city? We would like to see the results. Third, even if participants are perfectly balanced between the clusters, this does not preclude the use of multilevel analysis. This would still be extremely useful to know what proportion of the unexplained variance is attached to which level.

We thank you for this comment the Reviewer for this critical statistics-related comment.

As suggested, in order to determine a potential role played by the Administrative Sector (level1) and household (level2), we ran a multilevel analysis. Only the implementation ofPoisson regression resulted in converging. We did consider that even though results were obtained for Emergency and non-severe conditions. For severe conditions, it failed to converge, possibly due to effects of the data complex structure or overparameterization (Bates, 2015). A supplementary Table (S1) is provided in the manuscript. As a result, no complete comparative feature could be done with the GEE analysis.

**Reviewer #2**:

The author has addressed many of the previous reviewers' comments. However, there are still a few to be attended to. These are in highlights and notes in the revised manuscripts.

The sample size and sampling procedure needs to be made clearer and more details provided.

We thanks the reviewer for the comment.

As recommended, we provided more details regarding the sample size determination and sampling procedure.
A few typo and grammar still need to be addressed

We thank the reviewer for the comment.

The revised manuscript was proofread by two native speakers coauthoring the paper.

The title of the abstract does not have “of” inserted after “context”

 The comment was addressed. The “of” is inserted after “context”
