# Supplementary material for: Factors associated with health-seeking behavior amongst children in the context of free market: Household study in Ouagadougou, Burkina Faso, 2011
Source: PLoS One. 2022 Oct 18;17(10):e0271493. doi: 10.1371/journal.pone.0271493 (PMC9578640; doi:10.1371/journal.pone.0271493)
Supplement: Attachment — Submitted filename: review comments.docx [file pone.0271493.s004.docx]

Paper: Urban children health-seeking behavior in the context of free market: Household study in Ouagadougou, Burkina Faso

1. The title is very broad and year is missing. I would suggest a title “A cross-sectional study on health seeking behaviour amongst children from urban Ouagadougou in Burkina Faso: 2011” or “Factors associated with health seeking behaviour amongst children from urban Ouagadougou in Burkina Faso: 2011”
2. The data looks very old and outdated as it is almost 10 years older. The HSB may have changed in Ouagadougou in Burkina Faso.
3. In the abstract the authors do not come out very clearly about what conditions they are investigating. For example the following two papers (<https://www.ncbi.nlm.nih.gov/pmc/articles/PMC8364791/> or <https://onlinelibrary.wiley.com/doi/full/10.1111/tmi.13499> ) are clear on what was being investigated and what new knowledge they are contributing to HSB. I would recommend citation of <https://onlinelibrary.wiley.com/doi/full/10.1111/tmi.13499> by the authors since this is amongst the few papers focusing on many diseases amongst the children.
4. The authors used GEE but a random effects model is better choice.
5. The introduction is too long. The authors should reduce it to two or three paragraphs
6. This study is more of secondary data analysis from the original study done in 2011, the methodology has been written as though the authors recently conducted the study design. The authors should reflect the secondary data analysis aspect of their study and revise the methods in that respect.
7. Health seeking behaviour is not clearly defined in the paper. The authors should consider reading this paper: <https://onlinelibrary.wiley.com/doi/full/10.1111/tmi.13499>
8. The data came from a complex survey but the data analysis section talks nothing on how complex design was taken into account in the analysis.
9. Why did the authors perform the analysis in three different software? Just one software suffices.
10. The analysis section does not specify the statistical model was used in the “GEE” yet the results present ORs
11. It is also not clear how the regression models were arrived at although there is a mention of sensitivity analysis
12. What was the level of statistical significance?
