# Supplementary material for: Factors associated with health-seeking behavior amongst children in the context of free market: Household study in Ouagadougou, Burkina Faso, 2011
Source: PLoS One. 2022 Oct 18;17(10):e0271493. doi: 10.1371/journal.pone.0271493 (PMC9578640; doi:10.1371/journal.pone.0271493)
Supplement: Attachment — Submitted filename: Response to Reviewers Comments - Plos One-F.docx [file pone.0271493.s005.docx]

**Response to Reviewers**

**Editorial office**

**Comment:**

**1.** Please ensure that your manuscript meets PLOS ONE's style requirements, including those for file naming. The PLOS ONE style templates can be found at

https://journals.plos.org/plosone/s/file?id=wjVg/PLOSOne_formatting_sample_main_body.pdf and

https://journals.plos.org/plosone/s/file?id=ba62/PLOSOne_formatting_sample_title_authors_affiliations.pdf

**Response:**

Done.

**Comment:**

**2.** Thank you for stating the following in the **Acknowledgments Section** of your manuscript:

This paper is part of a doctoral project for which the author received a fellowship from Taiwan-ICDF and a research grant from the International Health Program of National Yang Ming University.

**Response:**

Done according to your suggestion.

**Comment:**

We note that you have provided additional information within the Acknowledgements Section that is not currently declared in your Funding Statement. Please note that funding information should not appear in the Acknowledgments section or other areas of your manuscript. We will only publish funding information present in the Funding Statement section of the online submission form. 

**Response:**

Done according to your suggestion.

**Comment:**

Please remove any funding-related text from the manuscript and let us know how you would like to update your Funding Statement. Currently, your Funding Statement reads as follows:

The funders had no role in study design, data collection and analysis, decision to publish, or preparation of the manuscript.

**Response:**

The author received a fellowship from Taiwan-ICDF and a research grant from the International Health Program of National Yang Ming University. The publication fees were supported by the Faculty of Health Sciences, University of Ottawa. The funders had no role in study design, data collection and analysis, decision to publish, or preparation of the manuscript.

**Comment:**

Please include your amended statements within your cover letter; we will change the online submission form on your behalf.

**Response:**

This information is included in the cover letter

**Comment:**

**3.** Please include captions for your Supporting Information files at the end of your manuscript, and update any in-text citations to match accordingly. Please see our Supporting Information guidelines for more information: http://journals.plos.org/plosone/s/supporting-information.

[Note: HTML markup is below. Please do not edit.]

**Response:**

Done according to your suggestion.

**Reviewer #1**

**Comment (**Methods):
The question for asking the health seeking behavior: is it based on a hypothetical scenario or based on a real case?

**Response:**

Thank you for this comment.
As you can see in the data collection tool, the questions #8, #11, and #14 are framed like: Where does [name of the household member] seek care in case of emergency matter? (See the questionnaire). The questions were not framed in hypothetical perspective. Instead, we sought their usual health-care-seeking behaviour, including their source of care in the 3 scenarios (Emergency, Severe, and not severe).
The question #33. “Could you tell me if you have suffered from any disease/injury in the past 30 days?” was reframed toward the end in order to validate their statement.
Indeed, this still does not rule out the potential bias, the recall bias for instance.

**Comments:**

Have all children experienced all the conditions asked, the emergency, severe or non-severe?

Is there any question to make sure that the children have experienced all that conditions? Do you have data about the diseases that the children have had? Please elaborate more.

**Response:**
Thanks this important comment.

Yes, there was a refined question regarding an experience of disease.

However, we noted that about 540 children have experienced a disease condition. I published on that question (Beogo, I., Huang, N., Gagnon, MP. *et al.* Out-of-pocket expenditure and its determinants in the context of private healthcare sector expansion in sub-Saharan Africa urban cities: evidence from household survey in Ouagadougou, Burkina Faso. *BMC Res Notes* **9,** 34 (2016). https://doi.org/10.1186/s13104-016-1846-4).

As the question was asking any disease experience of the past 30 days, obviously, we could not expect them to experience all the three conditions in this time frame (30 days). That was the main motive to look for their health-seeking behavior in case any of the three conditions occurred.

**Comment:**

The authors write that: for the purpose of this paper, self-medication and traditional healers were aggregated into the single category "informal provider". Can you elaborate more, perhaps in the introduction, how the position of traditional providers in the health care system in SSA?

**Response:**
In the SSA health system, although traditional providers play an important role as a resort in health providing, they are not part of the formal health system, not plotted in the health pyramid.

An excerpt was added in order to offer more light to the reader (p. 5).

**Comments:**

Discussion:
Based on your data, can you discuss the differences between the pattern of caregiver’s HSB for children and the pattern of adults HSB? I see that you have published an article about the HSB for adults.
As in other country and cultural setting, the pattern for children HSB is different than that of adults as in this article:
Widayanti AW, Green JA, Heydon S, Norris P. Health-Seeking Behavior of People in Indonesia: A Narrative Review. J Epidemiol Glob Health. 2020 Mar;10(1):6-15. doi: 10.2991/jegh.k.200102.001.

**Response:**

As suggested, an excerpt was added to light more readers on the patterns of HSB for children versus adults.

Please see on page 17.

**Reviewer #2**

**Comments:**

**1.** The title is very broad, and year is missing. I would suggest a title “A cross-sectional study on health seeking behaviour amongst children from urban Ouagadougou in Burkina Faso: 2011” or “Factors associated with health seeking behaviour amongst children from urban Ouagadougou in Burkina Faso: 2011”

**Response:**

Thanks for your comment. As suggested, we include the period of the study (2011) in the study title.

We also took the opportunity of your comment to reshape the title in the light of the Burkina Faso context at the data collection period.

**Comments:**

**2.** The data looks very old and outdated as it is almost 10 years older. The HSB may have changed in Ouagadougou in Burkina Faso.

**Response:**

Yes, indeed, the number of privates facilities have augmented in Ouagadougou, since. However, no recent study has repeated the exercise. Therefore, we lack evidence to hypothesize, as the main indicators (e.g., poverty rate, 2014 in 2014 [See Kobiané, 2020]) have not change significantly.

Kobiané, J., Ouili, I. & Guissou, S. (2020). Etat des lieux des inégalités multidimensionnelles au Burkina Faso. Dans : Jean-François Kobiané éd., *État des lieux des inégalités multi-dimensionnelles au Burkina Faso* (pp. 1-89). Paris Cedex 12: Agence française de développement. [https://doi.org/10.3917/afd.zanfi.2020.01.0001"](https://doi.org/10.3917/afd.zanfi.2020.01.0001)

**Comments:**

**3.** In the abstract the authors do not come out very clearly about what conditions they are investigating. For example the following two papers (https://www.ncbi.nlm.nih.gov/pmc/articles/PMC8364791/ or https://onlinelibrary.wiley.com/doi/full/10.1111/tmi.13499 ) are clear on what was being investigated and what new knowledge they are contributing to HSB. I would recommend citation of https://onlinelibrary.wiley.com/doi/full/10.1111/tmi.13499 by the authors since this is amongst the few papers focusing on many diseases amongst the children. by the authors since this is amongst the few papers focusing on many diseases amongst the children.

**Response:**

Thanks for this fundamental inquiry.

In fact, this study question was not targeting a specific disease. In contrary to Ng’ambi et al. (2020a) and Ng’ambi et al. (2020b) data were collected on the providers sought versus the condition severity based on their past experience (past 30 days). However, the question focusing on many diseases amongst the children was exploited and published for both children and adult.

I found the papers quite interesting for ma manuscript and cited.

**Comments:**

**4.** The authors used GEE but a random effects model is better choice.

**Response:**

Thanks for this fundamental comment. Indeed, and intuitively, the two-stage sampling approach led to a statistical approach that takes into account the hierarchical structure of the data. The clustering design was set with the aim of powering the representativeness of the sample.

In order to determine a potential role played by the Administrative Sector (level1) and household (level2), we ran a multilevel analysis. In running the GEE, we considered the common choice of Unstructured Correlation to the three others. We paid a wise attention to the sensitive issue of the statistical plan throughout the study implementation. At the design stage we discussed and kept in touch with Dr Liu Chieh-Yu, a biostatician and coauthor of two of my papers; the last publication stemmed from the same macro-project on healthcare-seeking-behaviour. Furthermore, Dr Maxime K. Drabo, a domestic researcher was consulted and has actively contributed set the field method and validated the questionnaires before and after the pretest. After the completion of the data collection, we were so lucky to re-discuss and validate the statistical strategy with Prof Kung-Yee Liang ―Who was the principal of my University and beyond, has coined the GEE with Scott L. Zeger. The first paper published in PLOS ONE adhered to similar statistical mindset. https://journals.plos.org/plosone/article?id=10.1371/journal.pone.0097521

**Comments:**

**5.** The introduction is too long. The authors should reduce it to two or three paragraphs

**Response:**

Done accordingly. The revised introduction counts 259 words.

**Comment:**

**6.** This study is more of secondary data analysis from the original study done in 2011, the methodology has been written as though the authors recently conducted the study design. The authors should reflect the secondary data analysis aspect of their study and revise the methods in that respect.

**Response:**

This is a part of the PI Doctoral thesis. He collected primary data from 1600 households. That is way, in the methods, we kept the main then characteristics of the study setting. For instance, we inform about the research ethics grant and kept the then administrative sectors distribution. This may help the reader, namely of Burkina Faso, to be cautioned of the findings.

**Comment:**

**7.** Health seeking behaviour is not clearly defined in the paper. The authors should consider reading this paper: <https://onlinelibrary.wiley.com/doi/full/10.1111/tmi.13499>

**Response:**

Thanks for the suggestion. We found it very interesting and even used it to give more light on the HSB definition in the background.

**Comment:**

**8.** The data came from a complex survey but the data analysis section talks nothing on how complex design was taken into account in the analysis.

**Response:**

We thank you for this comment the Reviewer for this critical statistics-related comment.

As suggested, in order to determine a potential role played by the Administrative Sector (level1) and household (level2), we ran a multilevel analysis. Only the implementation of Poisson regression resulted in converging. We did consider that even though results were obtained for Emergency and non-severe conditions. For severe conditions, it failed to converge, possibly due to effects of the data complex structure or overparameterization (Bates, 2015).

An excerpt was inserted to illustrate the complexity of data structure the statistical plan.

Please refer to “***Statistical analysis”*** subsection (page 9).

**Comment:**

**9.** Why did the authors perform the analysis in three different software? Just one software suffices.

**Response:**

This was just for transparency matter as I started with SPSS and completed the analyses with Stata and SAS. In the present version we removed the Stata.

**Comment:**

**10.** The analysis section does not specify the statistical model was used in the “GEE” yet the results present ORs

**Response:**

Thanks for this comment. I have tried my best to address this question when answering to the comment 8.

**Comment:
11.** It is also not clear how the regression models were arrived at although there is a mention of sensitivity analysis

**Response:**

I am not clear with this question. However,

**Comment:**

12. What was the level of statistical significance?

**Response:**

I sorry that in this study I opted to not present the *p-value*. With my previous manuscripts, I used to meet reviewers who disagreed with *p-value* presentation. That is the only reason only the Confidence Interval is exhibited.

**Comment:**

**13.** Table 2 talks about severity of illnesses. The missing piece is what was the illness? It is very misleading to just focus on illness severity.

**Response:**

Thank you for this comment.
The study did not focus on a specific disease. The questions were not framed in hypothetical perspective. We sought their usual health-care-seeking behaviour, including their source of care in the 3 scenarios (Emergency, Severe, and not severe).
In order to validate that quiring approach, there was a refined question regarding an experience of disease in the past 30 days.

I published on that question (https://bmcresnotes.biomedcentral.com/articles/10.1186/s13104-016-1846-4).
