# Supplementary material for: Factors associated with health-seeking behavior amongst children in the context of free market: Household study in Ouagadougou, Burkina Faso, 2011
Source: PLoS One. 2022 Oct 18;17(10):e0271493. doi: 10.1371/journal.pone.0271493 (PMC9578640; doi:10.1371/journal.pone.0271493)
Supplement: Attachment — Submitted filename: Response to Reviewers Comments - Plos One 20220609.docx [file pone.0271493.s006.docx]

**Response to Reviewers**

**Reviewer #3**

**Comment:**

1. In the abstract section, it is not clear what the reference category is University-educated household heads are compared in lines 16 to 19 on page 13.

**Response:**

Thanks for your comment.

To improve the reading flow, we explicitly added the reference group, that is participants choosing public provider.

**Comment:**

2. On page 6 and line 9, the sample size formula is better described as “formula for estimating single proportion”, not “simple random sampling formula”.

**Response:**

Thanks for your comment.

Correction done accordingly.

**Comment:**

3. On page 6 and line 21, The word “retained” should be replaced with “selected”.

**Response:**

Correction done as suggested.

**Comment:**

4. On page 6 and line 21, the word “and” should be replaced with “or”.

**Response:**

Thanks for this comment.

I could not figure out why ‘or’ fits better. Nevertheless, to improve the structure of the sentence and clarify the idea, two sentences are formed.

**Comment:**

5. On page 13 and line 20, delete the phrase “In conclusion”.

**Response:**

Done as recommended

Thanks
